# Physiotherapy clinical educators’ perceptions of student fitness to practise

**DOI:** 10.1186/s12909-016-0847-2

**Published:** 2017-01-17

**Authors:** Kristin Lo, Heather Curtis, Jennifer L. Keating, Margaret Bearman

**Affiliations:** 1 Department of Physiotherapy, Monash University, Peninsula Campus Building B McMahons Road Frankston, Melbourne, VIC 3199 Australia; 2 Department of Physiotherapy, Caulfield Hospital, Alfred Health, 260 Kooyong Road, Caulfield, Melbourne, VIC 3162 Australia; 3 HealthPEER Monash University, Clayton Road Clayton, Melbourne, VIC 3168 Australia

**Keywords:** Clinical education, Fitness to practise, Policy, Strategies, Student support

## Abstract

**Background:**

Health professional students are expected to maintain Fitness to Practise (FTP) including clinical competence, professional behaviour and freedom from impairment (physical/mental health). FTP potentially affects students, clinicians and clients, yet the impact of supervising students across the spectrum of FTP issues remains relatively under-reported. This study describes clinical educators’ perceptions of supporting students with FTP issues.

**Methods:**

Between November 2012 and January 2013 an online survey was emailed to physiotherapy clinical educators from 34 sites across eight health services in Australia. The self-developed survey contained both closed and open ended questions. Demographic data and Likert scale responses were summarised using descriptive statistics. The hypotheses that years of clinical experience increased clinical educator confidence and comfort in supporting specific student FTP issues were explored with correlational analysis. Open text questions were analysed based on thematic analysis.

**Results:**

Sixty-one percent of the 79 respondents reported supervising one or more students with FTP issues. Observed FTP concerns were clinical competence (76%), mental health (51%), professional behaviour (47%) and physical health (36%). Clinicians considered 52% (95% CI 38-66) of these issues avoidable through early disclosure, student and clinician education, maximising student competency prior to commencing placements, and human resources. Clinicians were confident and comfortable supporting clinical competence, professional behaviour and physical health issues but not mental health issues. Experience significantly increased confidence to support all FTP issues but not comfort. Student FTP issues affects the clinical educator role with 83% (95% CI 75-92) of clinicians reporting that work satisfaction was affected due to time pressures, emotional impact, lack of appreciation of educator time, quality of care conflict and a mismatch in role perception. Educators also considered that FTP issues affect service delivery and impact on those seeking health care.

**Conclusions:**

Strategies to support student FTP have potential to positively impact on students, clinicians and clients. Collaboration between these stakeholders is required, particularly in supporting mental health. Universities are strategically placed to implement appropriate support such as communication support.

## Background

Clinical educators are responsible for both client outcomes and educating future health professionals. Clinical education occurs in an environment constrained by time for service delivery, human resource limitations and fiscal pressures. Challenges occur when students require more intensive support than anticipated due to factors that affect their fitness to practise (FTP).

According to Parker [1, 2], FTP encompasses 1) ‘clinical competence’, 2) ‘professional behaviour’ and 3) ‘freedom from impairment’. These qualities underpin the safe practices of self-regulating professionals. Student FTP issues have the potential to negatively impact on the clinical education role, educator’s work satisfaction and client care. Despite their pivotal role as professional ‘gatekeepers’, clinicians are often inadequately prepared for assessing and supporting performance issues [3, 4]. Challenges of supervising students who are underperforming are well recognised [5–8]. Bearman and colleagues [5] reported that physiotherapy clinical educators had few educational strategies when dealing with underperformers aside from more time, effort or feedback. Other health professional educators have reported experiencing negative emotions when students do not achieve satisfactory results including fear and anxiety, and doubts about their ability to be an effective educator [7].

Underperformance and fitness to practise are not exactly the same. For example, a broken arm is an impairment that may prohibit fitness to practise, but clinical educators may not regard this as underperformance per se.

An innovation of this study is its equal emphasis on three domains: clinical competence, professional behaviour and freedom from impairment.

### Clinical competence

In a qualitative study into clinical competence issues that affect physiotherapy students, Hayes et al. [6] emphasised the importance of feedback in attaining competence. Educators need to identify incompetent behaviours early and discuss these with students [6]. The resultant behaviour change may lead to positive clinical outcomes [6].

### Professional behaviour

Management of professionalism (after recognised breaches) has dominated FTP research [9–11]. Universities have policies and procedures to manage professionalism issues, however policies differ between universities. As clinical educators can supervise students from a number of universities, they can be confused about the sequence of expected actions in response to professionalism issues.

### Freedom from impairment

Freedom from impairment refers to the level of physical and mental health required to work as a health professional. Physiotherapy students and physiotherapists are at risk of sustaining work related musculoskeletal disorders, particularly in the first five years following graduation [12]. Mental health issues such as depression, anxiety, stress and burnout (collectively termed psychological distress) are also evident in health professional students [13]. In a systematic review of burnout in medical students, IsHak and colleagues [14] reported that prevalence of burnout across nine included studies ranged from 45 to 71%. Burnout has also been reported in physiotherapy students [15], new graduates [16] and experienced physiotherapists [17]. Symptoms of burnout include emotional exhaustion, depersonalisation (where students begin to hold negative attitudes towards their clients) and low personal achievement, with students feeling incompetent and dissatisfied with their accomplishments [18]. Flagging these impairment issues early may minimise the impact on future practice [19]. Strategies to decrease the incidence of physical and mental impairments in physiotherapy students and clinicians are warranted.

### Rationale for the study

Hrobsky and Kersbergen’s [7] qualitative review into the views of nursing preceptors regarding students who performed poorly in the practice environment indicates the need for university-based faculty to listen, support and follow up with clinical educators. The goal of this research was to *listen* to the perceptions of physiotherapy clinical educators, using a mixed methods approach. Based on their views, systems can be designed that deliver the required *supports* and *follow up*.

The overarching research question was: What are Australian clinical educators’ perceptions of supporting FTP issues in physiotherapy students? Within this framework we posed sub-questions:

- Which types of FTP issues are clinical educators most and least confident or comfortable in supporting? Given clinical educators may be confident to discuss FTP issues with students but they may not necessarily be comfortable to do so, data regarding confidence and comfort were gathered separately.
- Do years of clinical experience lead to greater clinical educator confidence or comfort in supporting specific student FTP issues? Can student FTP issues be avoided and if so, what are suitable strategies?
- What is the perceived impact upon work satisfaction, the educator role and client care?

## Methods

In Australia, clinical educators are registered physiotherapists with a dual role in service delivery and student supervision. They are employed by the health services. Each health service may offer placements to students across a number of universities. For this study, we included clinical educators directly responsible for assessing competency in the skills expected on completion of a student clinical placement. The majority of health services participating in this study utilised university funding to employ a student coordinator. Student coordinators are clinicians who specialise in clinical education, support other clinicians in their educator role, and act as a point of liaison between health services and the university. The universities also employ on-campus academics who support students during clinical placements.

### Study setting

Thirty-four sites across eight health services in Victoria, Australia, that accept physiotherapy students for clinical placement, were invited to participate in this study. These represent acute and sub-acute care, private and public services and rural and metropolitan sites.

### Design

This was a cross-sectional observational study that gathered data using surveys of anonymous physiotherapy clinical educators.

### Process

Following approval by the Monash University Human Research Ethics Committee (CF10/1321 - 2010000703), independent ethics approval was gained from participating health networks: Alfred Health (443/12), Barwon Health (12/135), Monash Health (12306 L) and Peninsula Health (RRR: 291012). Executive approval was gained from Ramsay Health care. Departmental approval was gained from Epworth HealthCare, LaTrobe Regional Hospital and West Gippsland Healthcare Group.

### Participants

To create respondent anonymity, health service managers distributed the study explanatory statement to all physiotherapy staff members via email. If staff were educators and consented, they followed a link to an online survey. In maintaining ethical requirements, this procedure precluded confirmation of email recipients, and receipt or tracking of undeliverable emails [20]. We were therefore unable to determine how many health service managers acted on the request in the email and distributed it to staff, or how many staff opened and considered the invitation to participate. Consequently potential responder numbers could not be predicted. However the number of people sent the email might be as few as 79 people (100% response rate) or a many as 266 (30% response rate). The survey was conducted from November 2012 to January 2013.

### Instrument development

An online survey was developed collaboratively by university academic staff (KL, JK) and a clinical representative (HC) as no validated instrument existed that met the needs of this evaluation. The survey questions were based on literature demonstrating high rates of burnout in physiotherapy clinicians and the consequences on their work satisfaction and client care [1]. Questions were designed to establish both the confidence and comfort of clinical educators across different levels of experience in supervising students with FTP issues. To refine the face-validity of the developed instrument, the survey was tested by experts (Monash University and Health Service physiotherapy staff) to gauge whether the questions effectively captured relevant information. Administrative staff also tested the survey. Feedback was requested on content for clarity, consistency, ease of use and on whether respondents used the scale appropriately. The survey was modified in response to written and/or verbal feedback. The full survey is presented in Table 1. In alignment with the research questions, the data relating to FTP issues (arising from responses to Questions 1-12) are reported here. The survey contained multiple choice, free text and questions with response options such as a 10 point Likert scale with verbal anchors from 1 (extremely non confident) to 10 (extremely confident). A 10-point numerically anchored scale was chosen for the items pertaining to clinical educator confidence and comfort to provide opportunities to record subtle differences in responses [21].

**Table 1:** Clinical educator demographics (*n* = 78)^a^, number of students supervised, number of students with FTP issues in 2012, types of FTP issues and support utilised

*Q1. Approximately, how many years have you been practising as a physiotherapist?* 0–1 year; >1-3 years; >3-5 years; >5-10 years; 10+ years	
*Q2. Approximately, how many students did you “supervise” (i.e. you were involved in marking the student’s APP) in 2012?* 0; 1; 2; 3; 4; 5;6–10; 11–15; >15	
*Q3. Approximately how many of these students had FTP issues?* For example clinical competence, professional behaviour, physical or mental health issues that impacted on a students’ ability to perform the functions of a clinician safely and effectively. 0; 1; 2; 3; 4; 5;6–10; 11–15; >15	
*Q4. In 2012, the students with FTP issues were managed* (grade each of the following using a 5 point Likert scale, 1 = never, 5 = always) alone +/− departmental staff members (including manager); with input from Health Service co-ordinator; with input from campus based university staff).	
*Q5. What are the signs that typically alert you to a potential FTP issue?* (free text).	
*Q6. What type of FTP concerns have your students experienced?* Select one or more of clinical competence; professional behaviour; physical health; mental health; other (please explain, free text)	
*Q7. Could any of the FTP issues have been avoided?* Yes; no; not applicable (no FTP concerns) If yes, how these might have been avoided (free text).	
*Q8. Can you give some examples of how FTP issues have impacted on client care?* (free text).	
*Q9. Can you give some examples of how student FTP issues impact on your work as a clinical educator?* (free text).	
*Q10. Do student FTP issues affect your work satisfaction? yes; no. If yes, in what way?* (free text)	
*Q11. How confident did you feel to manage the different types of FTP issues listed?* Rate each of the following using a 10 point Likert scale, 1 = extremely non confident to 10 = extremely confident: clinical competence; professional behaviour; physical health; mental health. Comment on the strategies used (free text).	
*Q12. How comfortable did you feel to manage the different types of FTP issues listed?* Rate each of the following using a 10 point Likert scale, 1 = extremely non confident to 10 = extremely confident: clinical competence; professional behaviour; physical health; mental health. Comment on the strategies used (free text).	

^a^Whilst 79 clinical educators responded, demographic data entered by 78 clinical educators

### Data analysis

Demographic data and Likert scale responses were summarised using descriptive statistics. Percentages and 95% confidence intervals were rounded to the nearest integer. Confidence intervals were calculated for yes/no item responses (Table 1). Given the data were from ordinal scales, we used Spearman’s rho, to test the relationship between clinician confidence and comfort. We also tested the hypotheses that clinical educator confidence and comfort in supporting specific student FTP issues might be related years of clinical experience. Comfort and confidence ratings for FTP issues related to clinical competence, professional behaviour, physical health and mental health were plotted against years of experience resulting in four correlations for each construct. As the comparisons for clinician confidence and competence were considered discrete scientific enquiries, each was considered independently in family-wise Bonferroni corrections. The α-level was subsequently adjusted using Bonferroni corrections to 0.0125 [22]. Data were analysed using GraphPad Prism® version 6.01.

Data in free text responses were coded by two independent researchers (KL and HC) and clustered into themes, using the principles of thematic analysis [23]. After consensus was achieved on the refined themes, the researchers independently re-coded data under each theme, using a frequency count as suggested for large data sets [24]. A second phase of consensus was conducted to check that responses were collated consistently under each theme. Illustrative quotes were then selected. Qualitative data were reported in order of appearance in the survey responses (Table 1). The number of clinical educator responses contributing to each theme is indicated in parentheses.

## Results

Seventy-nine surveys were returned. Participant demographics are summarised in Table 2.

**Table 2:** Participant demographics

Years practising physiotherapy	*n* (%)		
> 1–3	9 (12%)		
> 3–5	13 (17%)		
> 5–10	24 (31%)		
10+	32 (41%)		
Number of students supervised	*n* (%)		
0–1	10 (13%)		
> 1–3	9 (12%)		
> 3–5	19 (24%)		
> 5–10	33 (42%)		
10+	7 (9%)		
Number of students with FTP issues	*n* (%)		
0	29 (39%)		
1	26 (34%)		
2	13 (17%)		
3–5	9 (12%)		
Types of FTP concerns	*n* (%)		
Clinical competence	55 (76%)		
Mental health	37 (51%)		
Professional behaviour	34 (47%)		
Physical health	26 (36%)		
Other	0		
Support in managing FTP issues	Never – seldom *n* (%)	Half-time *n* (%)	Mostly-always *n* (%)
Alone +/− departmental staff members (including manager)	22 (38%)	8 (14%)	28 (48%)
With input from Health Service student coordinator	13 (25%)	11 (21%)	29 (55%)
With input from campus based university staff	20 (36%)	12 (21%)	24 (43%)

**Fig. 1:**
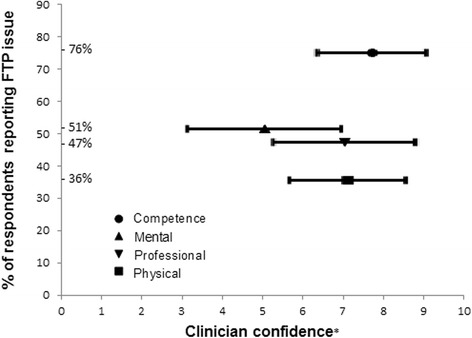
Clinical educator confidence in supporting FTP issues with *p*-values for tests for significant differences between FTP issues. Bonferroni adjusted α = 0.008. *VAS 0-10, higher scores = more confidence

The relationship between the reported incidence of each FTP issue and reported confidence in dealing with each FTP issue is shown in Fig. 1.

**Fig. 2:**
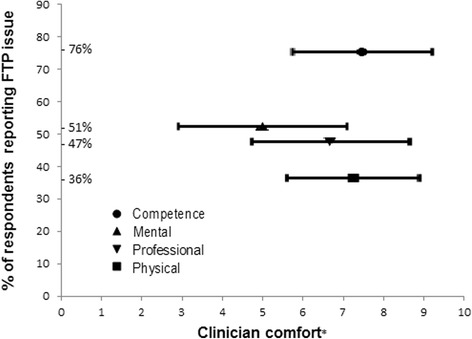
Clinical educator comfort in supporting different types of FTP issues. Bonferroni adjusted α = 0.008. *VAS 0-10, higher scores = more comfort

The relationship between reported FTP incidence and comfort in dealing with each issue is shown in Fig. 2.

Clinical educators appeared less comfortable supporting mental health issues compared with all other issues.

Moderate correlations were observed between confidence and comfort of (0.57 to 0.78) across all domains of FTP.

The correlation between greater confidence to manage FTP issues and more years of experience was significant: clinical competence (*r* = .61, *p* < 0.0001), professional behaviour (*r* = .49, *p* < 0.0001), physical health (*r* = .39, *p* = 0.0008), mental health (*r* = .42, *p* = 0.0003).

In contrast, the correlation between comfort with managing FTP issues and years of experience was not significant: clinical competence (*r* = .37, *p* = 0.019), professional behaviour (*r* = .30, *p* = 0.0117), physical health (*r* = .22, *p* = 0.065), mental health (*r* = .23, *p* = 0.0526).

### Signs that typically flag FTP issues

Seventy-one clinical educators responded to the free text question ‘What are the signs that typically alert you to a potential Fitness to Practise (FTP) issue?’ Analysis revealed the following five themes with the number of responses in parentheses:

- **Professional behaviours (43):** Over half of the clinical educators described issues with student professional behaviours e.g. “*overly laid-back attitude (*e.g. *poor attention to personal appearance, uniform, neatness, looking tired, bored, disinterested or inattentive, yawning frequently)”.*
- **Clinical competence (39):** Concerns regarding student competence with respect to a range of clinical skills e.g. *“Poor problems lists. Poor assessment skills”.*
- **Physical and mental impairments (39):** Student physical and mental impairment issues e.g. “… poor health”, “high stress levels, signs of emotional distress”.
- **Communication issues (16):** Communication issues such as culturally and linguistically diverse (CALD) factors e.g. *“poor communication with clients and colleagues”, “…poor English”.*
- **Recognising limits (10):** Issues with students overcommitting or not having insight into their own limitations e.g. *“failure to discuss complicated patients with senior staff where required”.*

### Strategies to prevent FTP issues

Of those clinical educators who had supervised students with FTP issues (*n* = 48/79), approximately half (25/48, 52%, CI = 38-66) felt that they could have been avoided and 23 (48%, CI = 34-62) felt that these were unavoidable. Thirty-three clinical educators responded to the free text question regarding how these FTP issues might have been avoided. Analysis revealed five themes:

- **Feed-forward (20):** Clinical educators were keen to be advised in advance about issues affecting student performance e.g. “if the student discloses this FTP issue to the university … before commencing the placement, it was much better”.
- **Student education (10):** Students need to be educated about FTP issues and their management e.g. “More education is required on what …the FTP issues are, and the student should feel safe to disclose…without fear of failing the placement”.
- **Clinical competency (9):** Strategies should be implemented to increase student clinical competency prior to attending clinical placements e.g. “Possibly improved training through the university in terms of risk assessment”.
- **Clinician education (5):** Clinical educators would benefit from additional professional development e.g. “We are not trained to deal with mental health issues!”.
- **Human Resources (3):** There are limitations in human resources e.g. “Given some educators have two students and a full (if not more) caseload, it can be easier to let fitness to practise issues slide”.

### Impact on client care

Sixty-four clinical educators responded to the free text question “Can you give some examples of how FTP issues have impacted on client care?” Clinical educators indicated that student FTP issues impacted on both student and educator client care, summarised in the following five themes:

- **Length of stay (36):** Student FTP issues impact on client length of stay e.g. “*Time efficiency and time management is compromised meaning patients are not receiving their therapy or discharge planning in a timely fashion without significant input from the supervisor*”.
- **Quality of care (30):** Quality of care is reduced as a result of student FTP issues e.g. “*Decreased quality of care due to a) student not providing best care b) supervisor spending increased time with student with FTP issues and that takes away from time with other patients”.*
- **Client experience (18):** The client experience is affected by student FTP issues e.g. “*Unable to perceive patients’ emotional reactions creating tension between patient and therapist, patient refusing therapy*”.
- **Safety (17):** There are safety implications associated with supervising students who have FTP issues e.g. “*Unsafe assessment and therefore treatment” including “risky transfers*”.
- **Professional behaviours (16):** Professional behaviours of students with FTP issues can be unacceptable e.g. “*Poor professionalism - not having clear professional boundaries with clients* e.g. *being too friendly, not being interested in the placement and only doing just enough to get by*”.

### Impact on clinical education role

Sixty-seven clinical educators responded to the free text question “Give some examples of how student FTP issues impact on your work as a clinical educator?” The following six themes were identified:

- **Clinical educator workload (39):** Student FTP issues affect clinical educator workload and overtime e.g. *“Many overtime hours have been spent either counselling or debriefing or waiting for students to finish their daily tasks”.*
- **Increased vigilance (36):** Increased attention is required when supervising students with FTP issues e.g. “*Require a lot more time and energy on my behalf, more phone calls to [the] university, more formal and informal feedback”*
- **Clinical educator wellbeing (20):** Student FTP issues affect clinical educator wellbeing e.g. *“Increased stress and feeling of responsibility towards bringing the students ‘up to scratch’” … “Clinical educator is taking stress leave from work due to the student FTP related issue”.*
- **Flow-on affects (19):** Students with FTP issues affect the health professional team e.g. *“As a stream we have needed to add in a lot more hands on tutes, to teach the students various topics that have not been covered by the universities”.*
- **Role change (7):** Clinical educators play a different role when supervising students with FTP issues e.g. *“I think the role of clinical educator can become less clinical and more of a social worker or psychologist. As this is not an area of expertise, this can leave clinicians in an unfamiliar environment”.*
- **Staff development (3):** The potential for further staff development to occur when students have FTP issues was discussed e.g. *“…senior staff always become involved, which can actually provide good learning experiences/mentoring for the supervisor”.*

### Impact on clinical educator’s work satisfaction

Of the 75 clinical educators who completed the question asking whether student FTP issues affected their work satisfaction, 62 (83%, CI = 75-92) stated ‘yes’ and 13 (17%, CI = 9-26) stated ‘no’. Most affected clinical educators (55) provided free text explanations. Responses were summarised in five themes:

- **Emotional response (34):** Clinical educators described affective responses to supervising students with FTP issues e.g. “*Most often, it makes my job less satisfying … However, when I have felt like I can help the student with these issues successfully and we have a good outcome, then my satisfaction … of my job is enhanced”.*
- **Time pressures (24):** Supervising students with FTP issues places time pressures on educators e.g. “*My patient caseload remains approximately the same with or without students. However, if a student has FTP issues a lot more time needs to be spent with [the student] leaving less time for everything else.”*
- **Lack of appreciation (11):** Educator’s experience lack of appreciation from students for clinical educator’s support e.g. *“ungrateful students.”*
- **Quality of care conflict (11):** There is a perceived conflict between supporting student education and providing quality client care e.g. “*I feel like the patient has not received good treatment and that impacts on job satisfaction …. Also sometimes (I) feel that FTP issues reflect my skills as a supervisor which impacts on work satisfaction and confidence*”.
- **Perception of roles (6):** Clinical educators’ changed their expectations of their role in clinical education e.g. “*Do not get to spend as much time as specialist physiotherapy educator and more time teaching basic life or communication skills*”.

### Clinical educator confidence in supporting FTP issues

In response to the question “How confident did you feel to manage the different types of FTP issues listed?” Thirty-two clinical educators provided free text descriptions of their strategies. Analysis revealed five themes:

- **Teaching strategies (24):** Use strategies to facilitate learning e.g. “*Encourage student to undertake reflective learning*”.
- **Proactive support (20):** Clinical educators actively sought support from colleagues e.g. “*Discuss with other clinicians about the student’s issues”,* “*Utilise university support staff early.”*
- **Open communication (15):** Open communication is important to educatorse.g. *“Try to allow open channels for them to talk to you about issues (identify the concern themselves)”.*
- **Mental health supports required (4)** Clinical educators requested assistance to support mental health issues e.g. “*We should not have to deal with mental health issues without help…”*
- **Documenting evidence (4):** Important to record examples of student performance e.g. “*Document examples of undesirable and desirable behaviours they demonstrate to draw on for feedback”.*

### Clinical educator comfort with supporting FTP issues

In response to the question “How comfortable did you feel to manage the different types of FTP issues listed?” Seventeen clinical educators provided free text descriptions of their strategies. Analysis revealed six themes:

- **Seeking support (9):** Clinical educators sought external advice in managing FTP issues e.g. *“My main strategy has been to discuss with as many other professionals as appropriate for advice on how to manage that particular issue”.*
- **Facilitating feedback (8):** It is important to engage students in targeted formal feedback e.g. *“Some students have required more organised formal feedback sessions, and structured/directed goal setting…”*
- **Clarifying and gathering information (6):** Set expectations and gather relevant information to manage FTP e.g. “*At the start of clinic set clear expectations of behaviour*”
- **Managing risk (3):** Clarify the process for identifying and managing risks to the students e.g. *“Mental health issues are always managed in conjunction with the university.”*
- **Joint supervision (2):** Supervision should be shared e.g. *“No one likes to fail a student…so these decisions are not taken lightly…Important to have at least one (other) PT (physiotherapy) staff supervising so that the decision is a joint decision”.*
- **Give the learner opportunity to respond to concerns (2)**: Give students the responsibility to respond to concerns e.g. *“Place onus on student to take some responsibility…”.*

## Discussion

This study provides an overview of FTP issues from the perspective of 79 physiotherapy educators. Experience with student FTP issues is common with 61% of the clinical educators surveyed reporting they had supervised one or more students with FTP issues in 2012 alone. Clinical educators reported experiences with all three elements of Parker’s [2] tripartite definition of FTP. While clinical competence was identified as an issue by 76% of the cohort, physical impairment was also identified by over 30% of the cohort. Mental health and unprofessional behaviours were also common experiences, with about half the cohort reporting experiences with these FTP issues. In addition to the tripartite definition, clinical educators reported that students’ with FTP issues may also be flagged by an inability to recognise their own limits, and communication issues.

This study produces two findings that are significant and add to the literature on this topic. Clinical educators proposed that physiotherapy students with FTP issues affect patient care. The most common effects were an increased length of stay (indicated 36 times) and a negative effect on the quality of care (indicated 30 times). This topic is under-explored in the literature, which tends to focus on impacts on educators. It is also a finding that deserves further research. Observational studies could triangulate this study, measuring the extent of this impact through additional measures and perspectives. It would also be interesting to determine whether this occurs in other health professions.

This study also identified that clinical educators perceive that 52% of student FTP issues can be avoided. This is significant in signalling the potential for additional strategies to support clinical educators. FTP literature typically focusses on consequences rather than prevention [9–11]. Respondents nominated “feed-forward” as a valuable strategy for prevention, that is, being provided with pre-existing information about learner needs in a timely manner to enable implementation of effective learning strategies to support students in the clinical environment. A debate exists regarding whether information about struggling students should be ‘fed forward’ to subsequent clinical educators due to potential for legal action, unfair treatment, bias and concerns for student privacy [3, 4, 25]. An option adopted by some physiotherapy programs is for students to complete a learning needs form prior to each clinical placement. This empowers students to articulate their educational needs while providing clinical educators with proactive strategies to facilitate learning. Additional approaches have involved student encouragement to self-disclose FTP issues to university academic staff prior to attending clinical placements and discuss potentially helpful strategies in advance with clinical educators to initiate timely support [26]. This enables students to feel comfortable while placing the onus on students to take responsibility for their learning. If FTP issues are known in advance, strategies such as peer learning and avoiding placement of two students with FTP issues with the same clinical educator could be utilised. Again, this is an area for future research. The role of peers in supporting FTP, as indicated by the frequency of referral to colleagues and university staff, also is worthy of exploration.

Other reported effects of supervising students with FTP issues, such as its impact on clinical educator capacity to complete their work, job satisfaction and burnout, align with previous studies. As reported by Bearman et al. [5] clinical educators tend to allocate more time, increase vigilance and undertake unpaid overtime to the expense of their wellbeing rather than developing alternative strategies [8]. This was evident in responses such as we “*needed to add in a lot more hands on tutes, to teach the students various topics that have not been covered by the universities*”. A long-term plan might involve key stakeholders in an iterative process of curriculum evolution with potential online resources to minimise the impact on human resources. In contrast to Bearman et al., clinical educators were aware of the importance of “*individualised and targeted*” strategies with a number of descriptions regarding shifting responsibility to the student. Of note was the importance of creating an environment that supports open communication about potential FTP issues without the fear of negative repercussions.

In alignment with previous literature [5, 7] clinical educators also voiced doubts about their ability as an educator when students were underperforming. While clinical educators stated that it could be stressful “*bringing the students up to scratch*” supervising students with FTP issues can provide a beneficial learning experience and an opportunity for educator mentoring.

Clinical educators generally feel confident and comfortable in addressing clinical competence issues but appear less confident and comfortable to manage mental health issues than any other FTP issue. This indicates the need for physiotherapy clinical educators to have accessible strategies to identify student mental health issues and respond appropriately including referral to appropriate support services. In randomised controlled trials, a mental health first aid program improved participant’s knowledge about mental health, increased confidence in helping those with mental health issues and benefitted participants’ own mental health [27, 28]. A mental health first aid program may be worth testing for relevance to both students and clinical educators.

Increasing years of experience significantly increased clinical educators’ confidence in supporting the spectrum of FTP issues. There was, however, no significant correlation between years of experience and comfort in dealing with FTP issues. This suggests that while clinical educators may grow in confidence, supporting these issues remains uncomfortable. The clinical educators’ suggestions of joint supervision and seeking proactive support from colleagues, student coordinators and university staff may be particularly beneficial for less experienced clinicians. There are opportunities for developing more sophisticated training programs that focus on obstacles to comfort such as a pathway approach or creating more awareness of what is achievable. However, while better strategies to improve clinical educator experiences may be put in place, it may also be important to recognise the inherent stress in supporting mental health issues, patient safety and other high stakes concerns.

### Implications for practise

Opportunities exist to expand on these findings to develop alternative strategies for clinical educators, applying evidence based strategies for supporting students with FTP issues across each of the FTP domains. Given the incidence of student mental health issues, clinicians’ stress and concerns about dealing with mental health FTP issues, there is an established need for mental health supports. The introduction of wellbeing curricula into entry level health programs has also shown positive results in terms of stress management and other indicators of mental health [29]. These programs might be extended to clinical educators.

### Limitations

There are limitations to this study, as with any survey research. The survey instrument was piloted but not formally validated. As we are unaware of the total number of invited participants, the response rate could not be calculated. In addition, those who responded to the survey may not be representative of those who did not respond and therefore it is not known how representative these views are of the population or of the setting in which clinical educators worked. Seventy-two percent of respondents had greater than 5 years’ experience and thus views may not represent less experienced therapists’ perceptions. This was an Australian study and physiotherapy specific. Confidence and comfort supporting individual FTP issues may differ according to discipline as physiotherapists have professional expertise in supporting physical impairment issues while social workers’ or psychologists have expertise in supporting mental health issues.

## Conclusions

Many physiotherapy students present with FTP issues. These appear to impact on client care, the role of the clinical educator and the work satisfaction of clinical educators. Clinical educators utilise a number of strategies to support students with FTP issues. Student support might benefit from improved processes to feed-forward students’ needs, develop skills to manage mental health issues when they arise and specific education of both students and clinical educators regarding FTP management. Additional research into evidence-based strategies to support student FTP is indicated.

## Abbreviations

FTP: Fitness to practise encompasses 1) ‘clinical competence’, 2) ‘professional behaviour’ and 3) ‘freedom from impairment’ [1, 2]
